# Astrocyte elevated gene-1 regulates astrocyte responses to neural injury: implications for reactive astrogliosis and neurodegeneration

**DOI:** 10.1186/1742-2094-9-195

**Published:** 2012-08-11

**Authors:** Neha Vartak-Sharma, Anuja Ghorpade

**Affiliations:** 1 University of North Texas Health Science Center, 3500 Camp Bowie Blvd, Fort Worth, TX, USA

**Keywords:** AEG-1, Astrocyte, HIV-1, Reactive astrogliosis

## Abstract

**Background:**

Reactive astrogliosis is a ubiquitous but poorly understood hallmark of central nervous system pathologies such as trauma and neurodegenerative diseases. *In vitro* and *in vivo* studies have identified proinflammatory cytokines and chemokines as mediators of astrogliosis during injury and disease; however, the molecular mechanism remains unclear. In this study, we identify astrocyte elevated gene-1 (AEG-1), a human immunodeficiency virus 1 or tumor necrosis factor α-inducible oncogene, as a novel modulator of reactive astrogliosis. AEG-1 has engendered tremendous interest in the field of cancer research as a therapeutic target for aggressive tumors. However, little is known of its role in astrocytes and astrocyte-mediated diseases. Based on its oncogenic role in several cancers, here we investigate the AEG-1-mediated regulation of astrocyte migration and proliferation during reactive astrogliosis.

**Methods:**

An *in vivo* brain injury mouse model was utilized to show AEG-1 induction following reactive astrogliosis. *In vitro* wound healing and cell migration assays following AEG-1 knockdown were performed to analyze the role of AEG-1 in astrocyte migration. AEG-1-mediated regulation of astrocyte proliferation was assayed by quantifying the levels of cell proliferation markers, Ki67 and proliferation cell nuclear antigen, using immunocytochemistry. Confocal microscopy was used to evaluate nucleolar localization of AEG-1 in cultured astrocytes following injury.

**Results:**

The *in vivo* mouse model for brain injury showed reactive astrocytes with increased glial fibrillary acidic protein and AEG-1 colocalization at the wound site. AEG-1 knockdown in cultured human astrocytes significantly reduced astrocyte migration into the wound site and cell proliferation. Confocal analysis showed colocalization of AEG-1 to the nucleolus of injured cultured human astrocytes.

**Conclusions:**

The present findings report for the first time the novel role of AEG-1 in mediating reactive astrogliosis and in regulating astrocyte responses to injury. We also report the nucleolar localization of AEG-1 in human astrocytes in response to injury. Future studies may be directed towards elucidating the molecular mechanism of AEG-1 action in astrocytes during reactive astrogliosis.

## Background

Astrocytes, as the most abundant cell type in the brain, respond to all forms of central nervous system (CNS) pathologies, from infection, injury and ischemia to neurodegenerative diseases, by a process commonly referred to as reactive astrogliosis. During this process, astrocytes undergo spectrum of changes in their molecular expression patterns and morphology, leading to both beneficial and detrimental effects on surrounding neural and non-neural cells. Despite the ubiquitous presence of reactive astrocytes at all sites of CNS pathologies, the molecular mechanisms, functions and effects of reactive astrocytes are surprisingly poorly understood and their roles in specific disease processes are largely unclear. Astrocyte elevated gene-1 (AEG-1), a novel human immunodeficiency virus (HIV)-1 and tumor necrosis factor α (TNFα)-inducible transcript, was identified in primary human fetal astrocytes in response to HIV-1 or TNFα treatment [1]. Subsequent to its identification, several studies have recognized AEG-1 as an oncogene, whose expression is elevated in many metastatic malignancies and is thereby implicated in cancer initiation, metastatic progression and chemotherapeutic resistance [2-6]. Although much is known about the role of this evolutionarily conserved gene in cancer [7], the role of AEG-1 in normal non-cancerous cells, such as astrocytes, has not yet been thoroughly investigated. In this report, we initiate studies to assess the role of AEG-1 in human astrocytes for mediating injury responses and further discuss their implications for HIV-1 CNS infection.

AEG-1 mRNA codes for a single pass transmembrane protein, with predicted molecular weight of 64 kDa, identified as a metastasis adhesion protein (metadherin) that enhances migratory capacities of cancer cells [8]. Despite the evolutionary conservativeness, the three-dimensional structure and functional motifs in the AEG-1 protein are still unresolved; however, several studies have reported multiple post-translational modifications, which might be responsible for the detection of this protein in varied intracellular compartments [9]. In the context of cancer, a transcriptional coactivator role has been attributed to AEG-1 [10] and multiple AEG-1-interacting proteins have been identified [10-13]. Subsequently, AEG-1 has been shown to localize to the cytoplasm, endoplasmic reticulum, nucleus and nucleolus in cancer cells [9]; however, its role in the different intracellular organelles, often considered a key determinant for its function, remain to be investigated, especially in normal non-cancerous cells. The initial discovery of AEG-1 in astrocytes following HIV-1 or TNFα treatment [1] implies its role in inflammatory responses of astrocytes. One of the major cellular manifestations of astrocyte inflammatory responses is reactive astrogliosis, in which activated astrocytes undergo rapid proliferation, enhanced migration towards the site of inflammation and attempt to mitigate collateral damage by isolating the damaged area [14-16].

As a hallmark of CNS pathologies, reactive astrogliosis is frequently associated with CNS insults such as infection, trauma, ischemia, neurodegeneration and post-neurosurgical healing, commonly associated with the management of brain tumors [17-19]. Gliosis is accompanied with increased expression of inflammatory cytokines and growth factors by surrounding astrocytes, which initiates a paracrine loop of signaling, potentiating proliferation, recolonization and migration of surviving cancer cells from the primary site of origin to secondary sites [20-22]. Since 70% to 95% of tumors recur from the tissues proximate to resection margins, reactive astrogliosis appears to create a favorable environment for cancer recurrence [23-26]. Thus, reactive astrogliosis is a significant phenomenon determining the rate of complete tumor clearance following conventional therapeutic interventions. Interestingly, the physiological changes in astrocyte behavior, function and outcome during hyperproliferative disorders, such as astrogliomas, which express very high levels of AEG-1, resemble those that occur during reactive astrogliosis [27], suggesting a common denominator. In spite of AEG-1 expression being minimal in non-cancerous adult cells, AEG-1 is highly expressed during the early mouse embryo developmental period from E8.5 to E9.5, suggesting a role in neurogenesis [28]. As AEG-1 has been shown to enhance cancer cell migration and proliferation as an adhesion protein and by inducing survival signals, it is likely to modulate astrocyte injury responses and ultimately affect the gliosis process and neurogenesis post-glial scar formation. Therefore, taking into account the pleotropic effects of AEG-1 in cancers, here we investigate the role of AEG-1 in orchestrating astrocytic responses to injury.

In this study, we show that AEG-1 is required for migration and proliferation of injured astrocytes to the site of injury. Results from this study, for the first time, identify AEG-1 as a novel mediator of reactive astrogliosis, implicating its role in neuroinflammation, consistent with its original identification in neuroinflammatory disease, HIV-1 CNS infection.

## Methods

### Non-obese diabetic-severe combined immunodeficiency mouse model of brain injury

Male, 4-week-old NOD-C.B-17 SCID mice were purchased from the Jackson Laboratory (Bar Harbor, ME, USA). Animals were maintained in sterile microisolator cages under pathogen-free conditions in accordance with the ethical guidelines for laboratory animals as set forth by the National Institutes of Health. All animal manipulations, including stereotactic injections, were performed in a laminar flow hood. Briefly, the animal was anesthetized and placed in a stereotaxic apparatus (Stoetling, Wood Dale, IL, USA) and intracranially injected as previously described [29]. Briefly, the animal’s head was secured with ear bars and mouthpiece. A 10 μl syringe injector was used to deliver a total of 5 μl of phosphate buffered saline (PBS) into the left hemisphere of the animal and the contralateral right hemisphere was used as control. Mice were killed 4 days post injection. Each brain was formalin fixed, embedded in paraffin and cut into 5 μm sections. The sections through the needle tract were identified by staining every eighth section with glial fibrillary acidic protein (GFAP) and 4',6-diamidino-2-phenylindole (DAPI, Life Technologies, Carlsbad, CA, USA). Adjacent sections surrounding the needle tract were stained by immunohistochemistry with primary antibodies specific to AEG-1 (1:100, rabbit monoclonal, Life Technologies) and GFAP (1:200, chicken polyclonal, Covance Inc., Emeryville, CA, USA) and Alexa Fluor® secondary antibodies, anti-rabbit (488 nm, green) and anti-chicken (594 nm, red) (1:100, Life Technologies), respectively. DAPI (1:800) was used to visualize the nuclei. Fluorescent images were visualized with an ECLIPSE Ti-4 using the NLS-Elements BR. 3.0 software (Nikon, Melville, NY, USA).

### Isolation and cultivation of primary human astrocytes

Human astrocytes were isolated from elective abortus specimens procured in full compliance with the ethical guidelines of the National Institute of Health, University of Washington and North Texas Health Science Center, as previously described [30]. Briefly, brain tissues were dissected and mechanically dissociated. Cell suspensions were centrifuged, suspended in media and plated at a density of 20 × 10^6^ cells/150 cm^2^ flasks. The adherent astrocytes were treated with trypsin and cultured under similar conditions to enhance the purity of replicating astroglial cells. The astrocyte preparations were routinely >99% pure as measured by immunocytochemistry staining for GFAP and microglial marker, CD68, to determine microglial content and possible contribution of microglia in inflammatory responses.

### RNA extraction and gene expression analysis

RNA was isolated (as described in [31]) from astrocytes treated as described in subsequent sections and gene expression was assayed by real-time polymerase chain reaction (PCR). TaqMan 5’ nuclease real-time PCR was performed using a StepOnePlus sequence detection system (Life Technologies). Commercially available TaqMan® Gene Expression Assays were used to measure AEG-1 (Life Technologies, cat. no. 4331182) and glyceraldehyde 3-phosphate dehydrogenase (GAPDH) mRNA levels (Life Technologies, cat. no. 4310884E). GAPDH a ubiquitously expressed housekeeping gene was used as internal normalizing control. The 30 μl reactions were carried out at 48°C for 30 minutes, 95°C for 10 minutes, followed by 40 cycles of 95°C for 15 s and 60°C for 1 minute in 96-well optical, real-time PCR plates.

### Immunofluorescent cytochemical analysis

Cultured human astrocytes were fixed, at experiment-specific time points, with 1:1 acetone: methanol (V/V) solution for 20 minutes at −20°C and blocked with blocking buffer (2% bovine serum albumin containing 0.1% Triton X-100) for 1 h. Cells were then incubated with primary antibodies specific to AEG-1 (1:200) or Ki67 (1:200, Abcam, Cambridge, MA, USA) or proliferating cell nuclear antigen (PCNA) (1:200, Cell Signaling Technologies, Danvers, MA, USA) and GFAP (1:400) in blocking buffer for 2 h. Cultures were then washed and stained with Alexa Fluor® secondary antibodies, anti-rabbit (488 nm, green), anti-mouse (488 nm, green) and anti-chicken (594 nm, red) (1:100). Nuclei were visualized with DAPI (1:800, Life Technologies). Micrographs were obtained on an ECLIPSE Ti-4 using the NLS-Elements BR. 3.0 software.

### Wound-healing assay

A well established *in vitro* wound-healing model previously characterized to study migration of cells in culture was utilized [32]. Briefly, human astrocytes were plated at a density of 0.1 × 10^6^ cells per 48-well or 2.0 × 10^6^ cells per 6-well tissue culture plate, and grown to confluence for 48 h. The medium was then aspirated and carefully, a thin stretch of the confluent layer was scratched using sterile 10 μl pipette tip to induce injury. Fresh astrocyte medium was added and the wound was allowed to heal. The wound healing and intracellular localization of AEG-1 in the migrating cells was evaluated at 8, 24, 36 and 48 h by fixing and performing immunostaining with anti-AEG-1 and anti-GFAP antibodies as described above. Live images of migrating cells were obtained using a phase contrast microscope (Zeiss Invertoscope 40C) (Carl Zeiss Microscopy, LLC, Thornwood, NY, USA) at 8, 24, 36 and 48 h. For protein analysis following wound healing, cells were plated to confluence into six-well tissue culture plates and injured by three equidistant parallel scratches/well, using a sterile 10 μl pipette tip. To ensure sufficient protein for analysis, multiple wells were scratched and combined protein pulled down for cytoplasmic and nuclear analysis.

### Confocal analysis

Human astrocytes were grown to confluence in glass bottom 48-well tissue culture plates (MatTek Corp., Ashland, MA, USA) at a density of 0.1 × 10^6^ cells/well for 48 h. The cells were then scratched, as described above, and carefully fixed and stained with antibodies specific to AEG-1, fibrillarin (1:200, mouse monoclonal, Abcam), GFAP and DAPI at 48 h post scratch. Micrographs were obtained on an Olympus IX71 Microscope (Olympus America Inc., Center Valley, PA, USA). Confocal colocalization analysis and two-dimensional histogram was performed using BioimageXD software (Free Software Foundation Inc., Boston, MA, USA). A *P* value >0.95 was accepted as statistically significant colocalization.

### siRNA transfection of astrocytes

Cultured human astrocytes were transfected with ON-TARGETplus® small interfering RNA (siRNA) specific to AEG-1 (siAEG-1), scrambled non-targeting siRNA (siCon, Thermo Scientific, Waltham, MA, USA) and without siRNA (mock) using the Amaxa^TM^ P3 primary cell 96-well nucleofector kit (Lonza, Walkersville, MD, USA) according to the manufacturer’s instructions. Briefly, 1.6 million astrocytes were suspended in 20 μl nucleofector solution containing siCon or siAEG-1 (100 nM) and transfected using a Nucleofector/Shuttle (Lonza) device. Transfected cells were supplemented with astrocyte media and incubated for 30 minutes at 37°C prior to plating. After 48 h, cells were washed and used experimentally.

### *In vitro* cell migration assay

The siRNA-transfected astrocytes were plated in 96 well Oris^TM^ cell migration assay plates (Platypus Technologies, Madison, WI, USA) with adherent cell-seeding stopper at a density of 7.5 × 10^4^ cells/well and incubated at 37°C for 48 h. Following growth to confluence, the cell-seeding stopper was removed and cells were washed with sterile PBS. Live cell imaging of cell migration into the stopper/wound zone was performed by treating cells in phenol-free astrocyte media with Hoechst, a nuclear dye (1:1,000, Life Technologies) at 37°C for 20 minutes prior to imaging daily for 5 days. Cell migration was quantified by counting the number of Hoechst positive cells in the center of the wound at each time point. Separate images from four replicate wells were assayed from 0 to 120 h and the average numbers of migrated cells were plotted. According to manufacturer’s instructions, an Oris^TM^ detection mask was kept intact to ensure the quantification of cells only in the detection zone (wound site).

### Measurement of cell proliferation

Non-transfected and transfected (mock, siAEG-1 or siCon) human astrocytes were plated in 48-well tissue culture plates for 48 h. The cells were fixed and immunostained for Ki67 or PCNA and GFAP as described above. Positive cells in ten micrographs of different quadrants in each of three replicate wells were counted.

### Western blot analysis

Confluent astrocyte cultures in six-well tissue culture plates were injured as described above. At 48 h after injury, cytoplasmic and nuclear protein extracts were isolated using the NE-PER nuclear and cytoplasmic extraction kit (Thermo Fisher Scientific). Cytoplasmic and nuclear protein samples (10 and 20 μg, respectively) were boiled with 4 × NuPAGE LDS loading sample buffer at 100°C for 5 to 10 minutes, resolved by NuPAGE 4% to 12% Bis-Tris gel and subsequently transferred to a polyvinyldifluoride (PVDF) membrane using i-Blot (Life Technologies). The membrane was incubated with anti-AEG-1 antibody (1:300) overnight at 4°C, washed and then incubated with anti-rabbit goat antibody IgG conjugated to horseradish peroxidase (1:10,000, Bio-Rad, Hercules, CA, USA) for 2 h at room temperature. The membrane was then developed with SuperSignal West Femto substrate (Thermo Fisher Scientific) and imaged in a Fluorochem HD2 Imager (Proteinsimple, Santa Clara, CA, USA). β-Actin (1:4,000, Sigma Aldrich, St. Louis, MI, USA), GAPDH (1:1,000, Santa Cruz Biotechonology, Santa Cruz, CA, USA) and Lamin A/C (1:3,000, Cell Signaling Technology) were used as loading controls for cytoplasmic and nuclear extracts, respectively.

### Statistical analyses

Statistical analyses were performed using Prism 5.0 (GraphPad Software, La Jolla, CA, USA), with one-way analysis of variance (ANOVA) and Newman-Keuls post test for multiple comparisons. Statistical analyses with a paired t test were used for comparisons between siCon and siAEG-1, and between no scratch and scratch. A two-way ANOVA was used for statistical analyses of comparison between siCon and siAEG-1 treatments. Significance was set at *P* <0.05 and data represents means ± SEM. Presented data are representative of a minimum of three independent replicates repeated in two or more independent donors.

## Results

### AEG-1 colocalizes with reactive astrocytes in an *in vivo* brain injury mouse model

**Figure 1:**
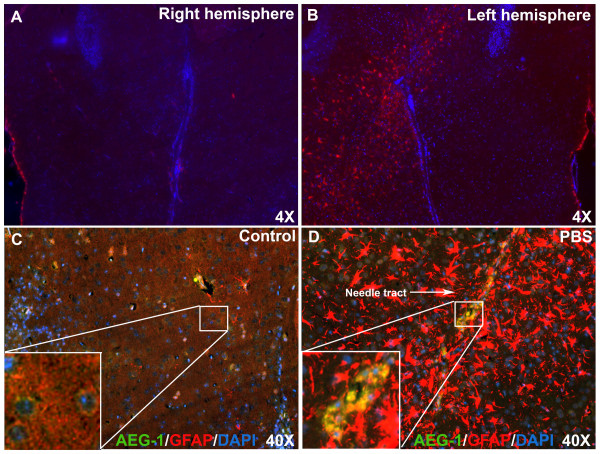
**Astrocyte elevated gene-1 (AEG-1) colocalizes with reactive astrocytes during astrogliosis.** AEG-1 expression in activated astrocytes of mouse brain, 4 days post stereotactic phosphate-buffered saline (PBS) injection, was analyzed by immunofluorescent microscopy. The non-injected contralateral hemisphere was used as a control. Immunostaining for glial fibrillary acidic protein (GFAP) (red, astrocyte marker) and 4', 6-diamidino-2-phenylindole (DAPI) (blue, nuclear marker) identified the needle tract of activated astrocytes (**A**,**B**). Non-injected (inset image, (**C**) and PBS-injected contralateral hemispheres costained for AEG-1 and GFAP, were assessed for AEG-1 expression and colocalization with GFAP (yellow, inset image, (**D**).

Reactive astrocytes in surgically resected and inflammatory areas of brain show an altered phenotype with increased proliferation and migration by modulating the expression and function of significant intracellular molecules [33-35]. Therefore, in the present study we analyzed AEG-1 expression in astrocytes undergoing reactive astrogliosis. An *in vivo* brain injury mouse model of reactive astrogliosis was used to investigate the change in AEG-1 astrocyte expression in response to injury in an intact healthy CNS. The model circumvents one of the central caveats for biochemical analyses, cellular heterogeneity, in large batches of *in vitro* primary astrocyte cultures. A stereotactic PBS injection (5 μl) into the claudate/putamen in the left hemisphere of a NOD/SCID mouse was compared to the non-injected contralateral hemisphere (Figure 1A). Immunostaining with GFAP (red, astrocyte marker) antibody and DAPI identified activated astrocytes comprising a glial scar tissue around the needle tract, 4 days post injection. Overall the number of GFAP positive cells increased, indicating reactive astrogliosis (Figure 1B). Furthermore, AEG-1 (green) costaining localized to the perinuclear cytoplasmic region of astrocytes in the non-injected contralateral hemisphere (inset image, Figure 1C). However, the number of AEG-1-GFAP double positive astrocytes (yellow, inset image, Figure 1D) increased dramatically near the needle tract. Thus, indicating that brain injury/trauma induces AEG-1 expression in astrocytes during reactive astrogliosis.

### AEG-1 regulates astrocyte migration and proliferation

**Figure 2:**
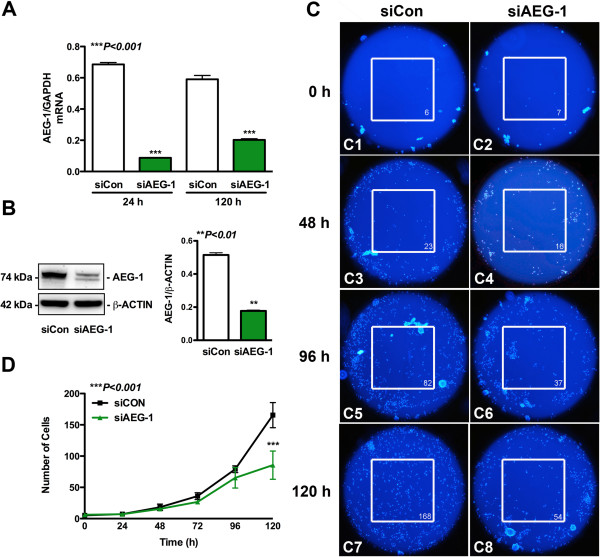
**Astrocyte elevated gene-1 (AEG-1) mediates astrocyte migration during wound healing.** Human astrocytes were transfected with AEG-1 specific siRNA (siAEG-1) or non-targeting, scrambled siRNA (siCon) by nucleofection and plated for 48 h. Messenger RNA was isolated at 24 h and 120 h post recovery and AEG-1 levels were measured by real-time polymerase chain reaction (PCR) (***P <0.001, (**A**)). In parallel experiments, immunoblotting was performed for AEG-1 at 48 h; β-actin was used as normalizing loading control (**P <0.001, (**B**)). Transfected astrocytes were plated to confluence for 48 h into Oris^TM^ migration assay plates and then injured by removal of the cell-seeding stopper. Migrating astrocytes were visualized with Hoechst nuclear stain at various time points. Micrographs are shown for siCon-transfected (**C1**,**C3**,**C5**,**C7**) and siAEG-1-transfected astrocytes (**C2**,**C4**,**C6**,**C8**). The number in the lower right corner of the white box (C1-C8) show number of cells in the area of the injury. Separate images from four replicate wells were analyzed to quantify the number of cells present (***P <0.001, (**D**)). Representative data from three individual donors assayed in triplicate is shown.

For further mechanistic studies involving the quantification of the dependency of astrocytes on AEG-1 for orchestrating cellular responses to trauma, we performed *in vitro* studies following AEG-1 knockdown in human astrocytes. This model allows direct manipulation of astrocyte AEG-1 responses and further characterization of its role during injury and inflammation. In order to investigate the role of AEG-1 in astrocyte migration, human astrocytes transfected with siAEG-1 and siCon were assayed by *in vitro* Oris^TM^ cell migration assay. Small interfering RNA specific towards AEG-1 significantly reduced AEG-1 mRNA levels at 24 h, which were sustained through 120 h (*P* <0.001, Figure 2A), and reduced 48 h AEG-1 protein levels by approximately three-fold (*P* <0.01, Figure 2B) as compared to siCon-transfected astrocytes. AEG-1 protein knockdown was sustained though 120 h (data not shown). In the Oris^TM^ cell migration assay, a relatively clear cell-free zone of injury was achieved at the beginning of the assay following removal of the cell-seeding stopper, in both siAEG-1-transfected and siCon-transfected astrocytes, which showed progressive healing by migrating astrocytes over 120 h (representative images, white box, Figure 2C1-C8). Directional movement of injured astrocytes towards the wound site was noted over time via injury-directed orientation of astrocyte nuclei. The wound was completely healed by 120 h in siCon-transfected astrocytes as compared to siAEG-1-transfected astrocytes. AEG-1 knockdown reduced astrocyte migration into the injury site, which was significant at 120 h as compared to siCon (*P* <0.001, Figure 2D). Thereby indicating that AEG-1 is required for astrocyte migration and thus may regulate the progression of wound healing.

**Figure 3:**
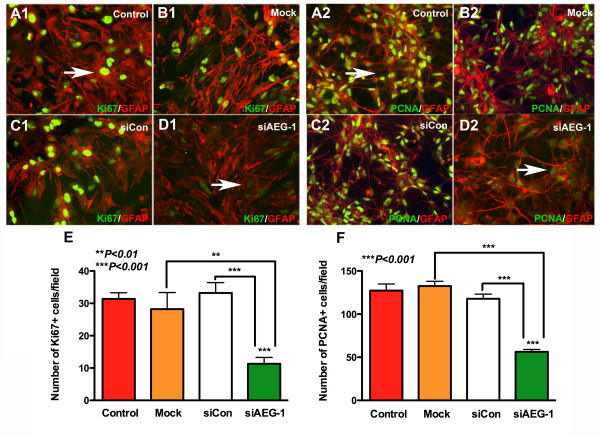
**Astrocyte proliferation requires astrocyte elevated gene-1 (AEG-1).** Astrocytes transfected with AEG-1 specific siRNA (siAEG-1) or non-targeting, scrambled siRNA (siCon) were immunostained with Ki67 or proliferating cell nuclear antigen (PCNA) (green, proliferation marker) and glial fibrillary acidic protein (GFAP) (red, astrocyte marker) antibodies. Untransfected and mock-transfected astrocytes were stained in parallel. Micrographs at 20 × original magnification of Ki67 or PCNA with GFAP costaining for non-transfected control (**A1**,**A2**), mock-transfected (**B1**,**B2**), siCon-transfected (**C1**,**C2**) and siAEG-1-transfected astrocytes (**D1**,**D2**) are shown. (**E**) This panel shows the number of Ki67 positive cells in ten micrographs from replicate wells for each condition (***P <0.001). (**F**) This panel shows the number of PCNA positive cells in ten micrographs from replicate wells for each condition (***P <0.001). All micrographs are 20 × original magnification, unless otherwise noted. Immunostaining data is representative of three individual donors assayed in triplicate.

The decrease in the number of Hoechst positive cells at the injury site following AEG-1 knockdown can also be accounted for by a change in astrocyte proliferation in addition to astrocyte migration. Therefore, to assess AEG-1 regulation of astrocyte proliferation, the expression of cell proliferation markers Ki67 and PCNA was assayed following AEG-1 knockdown in human astrocytes. Immunofluorescent staining for Ki67 (green, Figure 3A1-D1) or PCNA (green, Figure 3A2-D2) and GFAP (astrocyte marker, red) decreased in siAEG-1-transfected astrocytes as compared to controls. Nuclear Ki67 and PCNA staining was detected consistently in all controls. The Ki67 (arrow, Figure 3A1,D1) or PCNA (arrow, Figure 2A,D2) staining intensity as well as the number of Ki67 or PCNA positive astrocytes significantly decreased following AEG-1 knockdown as compared to controls (*P* <0.001, Figure 3E,F).These data demonstrate that astrocyte proliferation requires AEG-1.

### AEG-1 localizes to the astrocyte nucleolus during reactive astrogliosis

**Figure 4:**
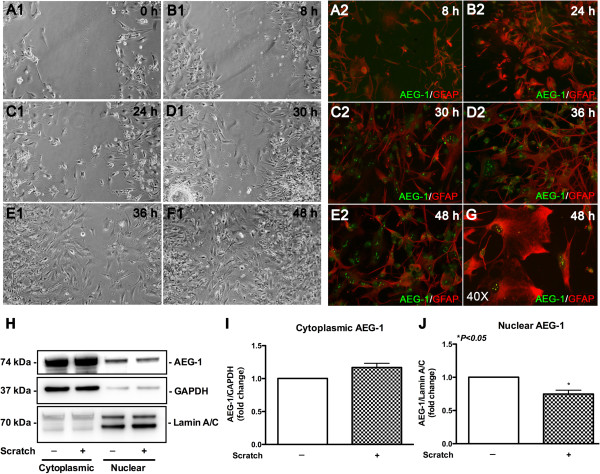
**Astrocyte elevated gene-1 (AEG-1) localizes to nuclear pockets during wound healing in cultured human astrocytes.** Confluent astrocyte cultures were scratched to mimic injury and subsequent wound healing by migrating astrocytes was monitored for up to 48 h. Phase-contrast images of the wound tract are shown from 0 to 48 h (**A1**-**F1**). In parallel, starting from 8 h post injury, cells were periodically fixed and immunostained with glial fibrillary acidic protein (GFAP) (red, astrocyte marker) and AEG-1 (green) antibodies (**A2**-**E2**). Higher resolution micrograph of AEG-1 and GFAP costaining in migrated astrocytes is shown (40 ×, (**G**)). All images are 20 × original magnification, unless otherwise noted. Cytoplasmic and nuclear protein extracts of astrocytes following wound healing were immunoblotted for AEG-1 (**H**); cytoplasmic glyceraldehyde 3-phosphate dehydrogenase (GAPDH) and nuclear lamin A/C were used as subcellular fraction normalizing controls (*P <0.01, (**J**)). Representative data from three individual donors assayed in triplicate. Immunoblotting data is representative of three individual donors.

**Figure 5:**
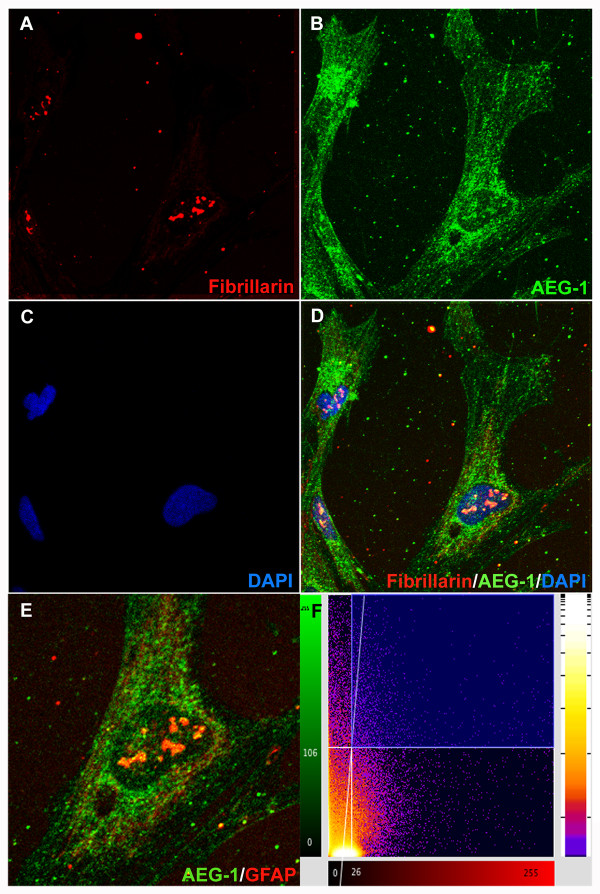
**Astrocyte elevated gene-1 (AEG-1) localizes to the astrocyte nucleolus during wound healing.** At 48 h post injury astrocytes were immunostained for fibrillarin (red, nucleolar marker, (**A**)), AEG-1 (green, (**B**)) and 4', 6-diamidino-2-phenylindole (DAPI) (blue, nuclear marker, (**C**)) and micrographed by confocal microscopy. Z-stack micrographs of fibrillarin fluorescence were overlaid upon AEG-1 and DAPI (**D**). Higher magnification micrograph of AEG-1 and fibrillarin costaining is shown (**E**). A scatter plot of overlapping AEG-1 and fibrillarin pixels was generated (*P* = 1, (**F**)). Representative confocal images are 200 × original magnification from 2 individual donors assayed in triplicate.

Next, we sought to identify possible injury-induced changes in the intracellular localization of AEG-1 in astrocytes using an *in vitro* wound-healing model. In this model, live phase contrast images of the wound showed complete healing by 48 h (Figure 4A1-F1). Migration of astrocytes into the wound site from either sides of the scratch/wound could be detected starting from 24 h. In response to injury, astrocyte morphology was altered with extensive processes protruding from their soma towards the wound site with dense staining for GFAP (red) and altered AEG-1 (green) intracellular localization (Figure 4A2-E2). Injury-induced activation of astrocytes was observed clearly in GFAP micrographs at 24 h (Additional file 1). Interestingly, AEG-1 was also detected in areas within the migrating astrocyte nucleus, which showed an increase in intensity in a time-dependent manner. Upon complete wound healing (48 h), AEG-1 clearly localized to nuclear pockets of migrated astrocytes (Figure 4G). These five to six dense AEG-1 positive areas in the nucleus of injured astrocytes differed significantly from the cytoplasmic and nuclear localization in non-wounded astrocyte cultures where AEG-1 appears more prominent in the perinuclear region, less so in the nucleoplasm, and is diffuse throughout the cytoplasm (Additional file 2). The injury-induced changes in AEG-1 intracellular localization were quantified by immunoblotting cytoplasmic and nuclear extracts of injured astrocytes for AEG-1 (Figure 4H). Injury induced higher cytoplasmic AEG-1 levels and significantly reduced nuclear AEG-1 levels as compared to non-injured astrocytes (Figure 4I,J). However, immunoblotting of astrocyte whole cell lysates, 48 h after injuries of increasing severity, did not indicate changes in AEG-1 protein levels (Additional file 3). The interesting observation of subnuclear AEG-1 being detected in injured astrocytes hinted towards nucleolar localization and initial immunocytochemical studies showed AEG-1 colocalization with nucleolin within the nuclei of astrocytes during wound healing (Additional file 4). The injury-induced specific subnuclear compartmentalization of AEG-1 in astrocytes was thus assayed by immunostaining for fibrillarin (nucleolar marker, red, Figure 5A) and AEG-1 (green, Figure 5B). Analysis revealed significant colocalization between fibrillarin and AEG-1 (*P* = 1.0; Figure 5C-F), signifying movement of AEG-1 to the nucleolus during wound healing.

## Discussion

The present work identifies AEG-1 as a novel modulator of astrocyte injury responses by regulating astrocyte migration and proliferation. Our *in vivo* brain injury mouse model and *in vitro* cell migration assays provides evidence for the induction of AEG-1 expression during gliosis in injured astrocytes and a subsequent alteration in subcellular distribution consequent to injury. We also report the nucleolar localization of AEG-1 during astrocyte wound healing, which provides direction for future mechanistic studies.

Reactive astrogliosis, a prerequisite for CNS wound healing, orchestrates events that lead to glial scar formation, which is critical for maintaining CNS homeostasis [36]. While AEG-1 was first described as an HIV-1-inducible transcript in astrocytes [1], this is the first report that describes a critical role of AEG-1 in reactive astrogliosis, the first line of defense for any CNS injury [23]. The *in vivo* brain injury mouse model provides evidence for the induction of AEG-1 expression during gliosis, however, AEG-1 levels in injured astrocytes remained unchanged in the *in vitro* wound-healing model. This distinction may be due to the difference in the complexities of the two models. In the intact CNS brain injury model there are elevated levels of AEG-1-inducing agents, such as TNFα and interleukin 1β (IL-1β), secreted by other non-neural cells such as activated microglia [37], in contrast to the confluent layer of pure astrocyte cultures of the *in vitro* wound-healing model. AEG-1 induction at the wound site in the *in vivo* brain injury mouse model colocalized with reactive astrocytes, indicating a possible involvement of AEG-1 in the injury responses of astrocytes.

During gliosis, astrocytes undergo multitude of changes in their gene expression patterns, secretion profiles and morphological traits that lead to a reactive phenotype capable of mediating wound healing [17,38,39]. AEG-1 induction following injury can alter many intracellular signaling pathways, such as nuclear factor (NF)κB [8,10], Wnt [40], cyclic adenosine monophosphate [5,41], mitogen activated protein kinases (MAPK) [40] and phosphatidylinositol 3-kinases-AKT [42]. It is interesting to note that AEG-1 expression in astrogliomas has been shown to induce CXCL8 expression [8] and matrix metalloproteinase-9 production [5], both of which show a dramatic increase during gliosis [43,44]. Reactive astrogliosis is often associated with deregulated glutamate clearance, which is responsible for toxicity to neurons [45,46]. Interestingly, AEG-1 has been shown to suppress the expression of excitatory amino acid transporter-2 in astrogliomas [47-49]. Besides the altered secretory profiles of reactive astrocytes, structural changes such as elevated expression of GFAP, vimentin and nestin are observed during reactive astrogliosis [50]. These can also be modulated by AEG-1, by functioning as a coactivator for signaling molecules, such as NFκB and MAPK. AEG-1 has been reported to physically interact with NFκB p65 subunit and activate NFκB responsive genes, most of which are overexpressed during gliosis [10,51].

Recruitment of astrocytes to the site of injury is an important primary step for initiation of reactive astrogliosis [36]. Here, we report for the first time AEG-1-mediated regulation of human astrocyte migration. As a metastasis adhesion protein, increased cytoplasmic AEG-1 expression has been shown to promote augmented migration, invasion and metastasis of cancer cells such as neuroblastoma and malignant gliomas, facilitating anchorage-independent growth and survival in the secondary site [1,52]. The *in vitro* wound-healing assay utilized in this study allows characterization of the innate injury-induced subcellular changes in AEG-1 localization excluding the confounding factors present in an intact CNS. Here, we report higher cytoplasmic AEG-1 levels and significantly reduced nuclear levels following injury, which is similar to AEG-1 localization in highly invasive cancer cells [9]. While AEG-1 has been shown to mediate metastasis as an adhesion protein, the interacting counterpart on other cells has yet to be identified and further mechanistic studies are lacking [53]. However, AEG-1 was previously reported to induce matrix metalloproteinase production in glioma cells, thereby facilitating glioma invasion [5]. A study in breast cancer revealed that AEG-1 promotes epithelial-mesenchymal transition and enhanced migration by upregulating mesenchymal markers, downregulating epithelial markers, and inducing nuclear accumulation of NFκB [5,54]. It has been reported that ectopic expression of AEG-1 could augment anchorage-independent growth of non-tumorigenic melanocytes and immortalized astrocytes [42], whereas AEG-1 knockdown reduced cell viability and promoted apoptosis in prostate cancer cells [55].

Our study demonstrated reduced astrocyte migration following AEG-1 knockdown. The decrease in migratory capacities was sustained over 5 days indicating that AEG-1 may also have influenced astrocyte proliferation. Astrocytic hyperproliferation is yet another hallmark of reactive astrogliosis [27]. AEG-1 knockdown also reduced astrocyte proliferation as assayed by change in Ki67 and PCNA expression. Nuclear protein Ki67 is expressed in all cell cycle phases except G_0_, whereas nuclear protein PCNA levels are highest during the G1/S phase of the cell cycle [56]. Therefore, the decrease in both Ki67 and PCNA positive astrocytes following AEG-1 knockdown reported here implies G1/S phase cell cycle arrest following AEG-1 knockdown, indicating a plausible role of AEG-1 during these cell cycle phases, and further studies are warranted. It remains to be seen whether overexpression of AEG-1 will enhance the ability of astrocytes to participate in gliosis.

Although, a number of AEG-1-interacting proteins have been identified [10,11,13], a complete understanding of the biological functions and biochemical characteristics, particularly the molecular triggers for differential sub-cellular localization and its subsequent effects on cellular mechanisms remains elusive to date. AEG-1 localization to the nucleolus has been reported in a few isolated cancer types, but is most frequently detected either in the nucleoplasm or cytoplasm of many metastatic tumors [9,57]. Here, we report injury-induced colocalization of AEG-1 with nucleolar protein fibrillarin, a component of the small nuclear ribonucleoprotein complex that is required for processing of the pre-rRNA molecules [58,59]. This suggests a novel role of AEG-1 in pre-rRNA processing and assembly. Aberrant ribosome biogenesis leads to p53-dependent G1 arrest, and therefore is crucial for cell survival and proliferation [60-62]. Recently, cytoplasmic AEG-1 has been identified as a RNA-binding protein, which provides survival advantage to cancer cells under conditions of stress by blocking Rad51 nuclear accumulation [12,63]. Furthermore, AEG-1 has been shown to physically interact with Staphylococcal nuclease domain-containing protein 1, a component of the RNA-induced silencing complex assembly, and to regulate microRNA processing and function in hepatocellular carcinoma [12]. However, proteomic studies failed to identify a DNA-binding domain on the AEG-1 protein. Together these findings imply a potential role as a scaffolding protein of the small nuclear ribonucleoprotein complex mediating either transcription and/or processing of the pre-rRNA molecules. The role of AEG-1 in the nucleolus has not been investigated to date in either cancer or non-cancerous cells. Therefore, the current finding of AEG-1 colocalization with fibrillarin provides a physiological basis for future studies. Additional studies into the physical interaction between AEG-1 and nucleolar-processing complex components and mechanism of participation in pre-rRNA processing are necessary to determine the role of AEG-1 in astrocytic wound-healing responses.

Restricted HIV-1 infection of astrocytes contributes to HIV-1-associated neuropathies *via* multiple mechanisms, including reactive astrogliosis and glutamate excitotoxicity [64]. In conjunction to the previous findings on AEG-1, as a mediator of glutamate excitotoxicity, this study identifies yet another mechanism of AEG-1-mediated regulation of HIV-1-associated neuropathies, via regulation of reactive astrogliosis. In addition, we have previously reported that disruption of astroglial-neuronal interactions and secretion of neurotoxic cytokines and chemokines during astrogliosis can also contribute towards neuronal cell atrophy [65]. Yet another important molecular change during reactive astrogliosis is the increase production of reactive oxygen species such as nitric oxide, nitric oxide synthase, super oxide dismutase and glotathione [61-63]. Interestingly, previous studies on AEG-1 in glioma have revealed AEG-1-mediated protection against glocuse deprivation-induced cytotoxicity [49,64]. Also, AEG-1 increased the chemotherapeutic resistance of breast cancer cells by increasing the expression ot multidrug resistance gene 1, thereby increasing the efflux of toxic waste from the cells [53,65,66]. Hence, further studies on AEG-1-induced alterations in the astrocyte secretary profiles are currently ongoing.

The oncogenic Ha-Ras pathway has been implicated to induce AEG-1 expression in cancers [66]; however, the pathway involved in the induction of AEG-1 in normal non-cancerous cells such as astrocytes has yet to be investigated. We are currently investigating many of the molecular triggers and modulators of reactive astrogliosis as plausible inducers of AEG-1 in human astrocytes, including cytokines and growth factors, ischemia-associated mediators like hypoxia [67], glucose-privation, and mediators of innate immunity such as LPS [68] and other toll-like receptor agonists. Thus, additional works to elucidate the molecular pathways involved in AEG-1 induction in human astrocytes during reactive astrogliosis are warranted.

## Conclusions

**Figure 6:**
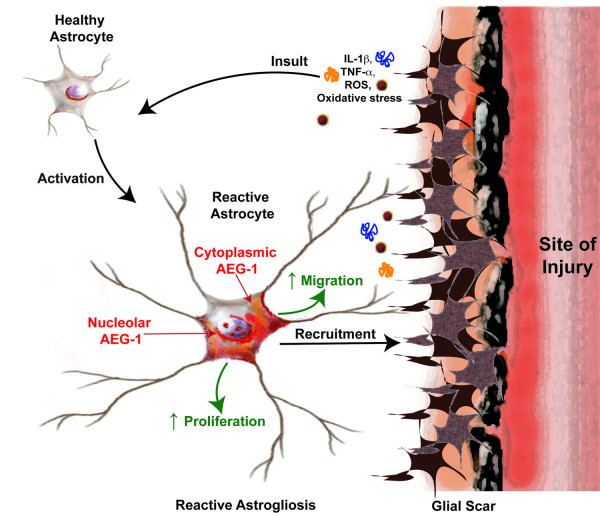
**Role of astrocyte elevated gene-1 (AEG-1) in reactive astrogliosis.** Central nervous system (CNS) insults ranging from mild cellular disturbances to severe tissue damage and cell death lead to release of molecular mediators of reactive astrogliosis such as inflammatory cytokines interleukin 1β (IL-1β), tumor necrosis factor α (TNFα) and molecules of oxidative stress such as reactive oxygen species (ROS). These mediators in turn activate the local healthy astrocyte population by inducing a spectrum of changes in the microenvironment and intracellular signaling pathways resulting into reactive astrogliosis. Injury triggers increased AEG-1 localization to the cytoplasmic regions and the dense fibrillar nuclear regions of the astrocyte. This change in intracellular localization of AEG-1 in astrocytes undergoing reactive astrogliosis is a plausible mechanism of AEG-1-mediated regulation of astrocyte proliferation and migration during reactive astrogliosis.

The present work identifies AEG-1 as a novel modulator of astrocyte injury responses by regulating astrocyte migration and proliferation. CNS insults of varying severities induce molecular mediators of reactive astrogliosis, which trigger induction and/or subcellular compartmentalization of AEG-1 protein in activated astrocytes to mediate enhanced migration and proliferation; thereby engendering the preliminary astrocytic responses to injury (Figure 6). The present finding of the potential function of AEG-1 in modulating the response of normal astrocytes surrounding injury site, can impact diverse outcomes in cancers, neuroinflammation and neurodegenerative diseases by determining the potential for tumor recurrence, healing of injuries as well as recovery from degenerative processes. Thus, the characterization of the involvement of AEG-1 during reactive astrogliosis has opened multiple avenues of investigations in neuropathology focused on the impact of surrounding astrocytes in regulating the response to disease progression as well as conventional therapeutic interventions.

## Competing interests

The authors declare that they have no competing interests.

## Authors’ contributions

NV-S: participated in conception, design and execution of all experiments. NV-S carried out data analysis and constructed the manuscript. AG: participated in conception, design and troubleshooting of all experiments. AG was essential in design and development of the manuscript. Both authors read and approved the final manuscript.

## Supplementary Material

Additional file 1 ***In vitro *wound-healing model induces reactive astrocytes at the wound site.** Confluent astrocyte cultures scratched to mimic injury were immunostained for glial fibrillary acidic protein (GFAP) to assay subsequent wound healing. At 24 h post injury, astrocytes near the wound site show dense GFAP staining with extensive protrusions arising from the stoma (white arrow) as against the relatively flat morphology of non-motile astrocytes away from the injury site (yellow arrow). Representative micrograph, 10 × original magnification, from three individual donors assayed in triplicate. Click here for file

Additional file 2 **Astrocyte elevated gene-1 (AEG-1) localizes to the cytoplasm and nucleus in cultured human astrocytes under unstressed conditions.** Human astrocytes were plated for 24 h in 48 well tissue culture plates at a density of 0.1 × 10^6^ cells/well. At 24 h post plating, cells were immunostained for AEG-1 (green, (A)), glial fibrillary acidic protein (GFAP) (red, astrocyte marker, (B)) and 4', 6-diamidino-2-phenylindole (DAPI) (blue, nuclear marker, (C)) and 20 × original magnification micrographs were overlaid (D). Cytoplasmic (50 μg/lane) and nuclear (20 μg protein/lane) protein extracts from astrocyte cultures were immunoblotted for AEG-1 (E). Representative data from three individual donors. Click here for file

Additional file 3 **Astrocyte elevated gene-1 (AEG-1) protein levels remained unchanged following *in vitro *injury to human astrocytes.** Human astrocytes were plated to confluence in six-well tissue culture plates and scratched to mimic injury using 10 μl pipette tips with increasing number of scratches (0, 1, 2, 3, 4, and 6). At 48 h post injury, whole cell lysates were immunoblotted for AEG-1 (A). β-Actin was used as normalizing control and normalized AEG-1 levels were plotted following band analysis (B). Representative data from two individual donors. Click here for file

Additional file 4 **Astrocyte elevated gene-1 (AEG-1) colocalizes with nucleolar protein nucleolin.** At 48 h post injury astrocytes were immunostained for AEG-1 (green, (A)), nucleolin (red, nucleolar marker, 1:200, Abcam, (B)) and 4', 6-diamidino-2-phenylindole (DAPI) (blue, nuclear marker, (C)) and 20 × original magnification micrographs were overlaid (yellow, inset, (D)). Representative data from two individual donors assayed in triplicate. Click here for file
