# Supplementary figures and images for: Astrocyte elevated gene-1 regulates astrocyte responses to neural injury: implications for reactive astrogliosis and neurodegeneration
Source: J Neuroinflammation. 2012 Aug 11;9:195. doi: 10.1186/1742-2094-9-195 (PMC3488579; doi:10.1186/1742-2094-9-195)

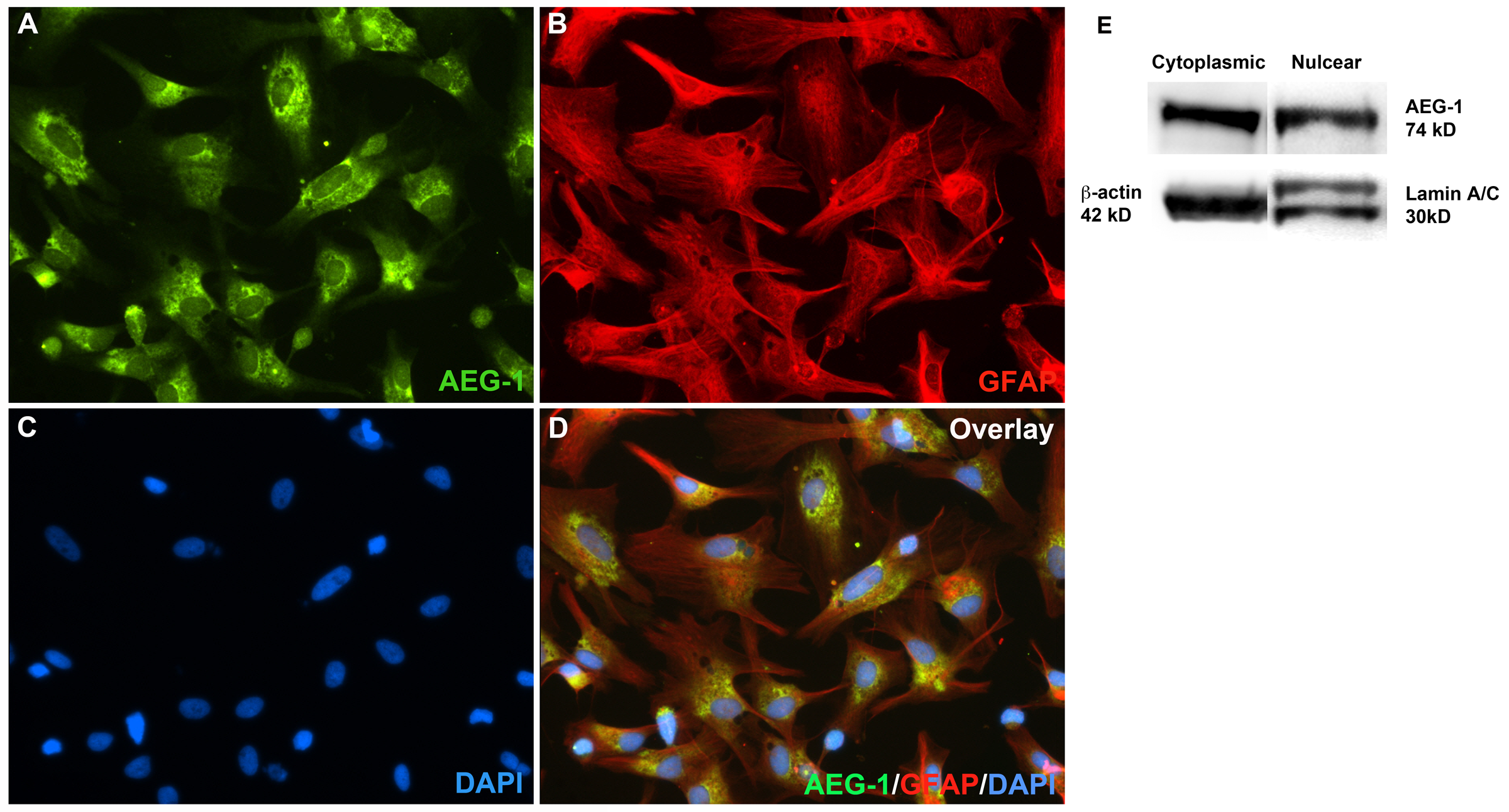

Supplement: Additional file 2 — Astrocyte elevated gene-1 (AEG-1) localizes to the cytoplasm and nucleus in cultured human astrocytes under unstressed conditions. Human astrocytes were plated for 24 h in 48 well tissue culture plates at a density of 0.1 × 10^6^ cells/well. At 24 h post plating, cells were immunostained for AEG-1 (green, (A)), glial fibrillary acidic protein (GFAP) (red, astrocyte marker, (B)) and 4', 6-diamidino-2-phenylindole (DAPI) (blue, nuclear marker, (C)) and 20 × original magnification micrographs were overlaid (D). Cytoplasmic (50 μg/lane) and nuclear (20 μg protein/lane) protein extracts from astrocyte cultures were immunoblotted for AEG-1 (E). Representative data from three individual donors. [file 1742-2094-9-195-S2.tiff]

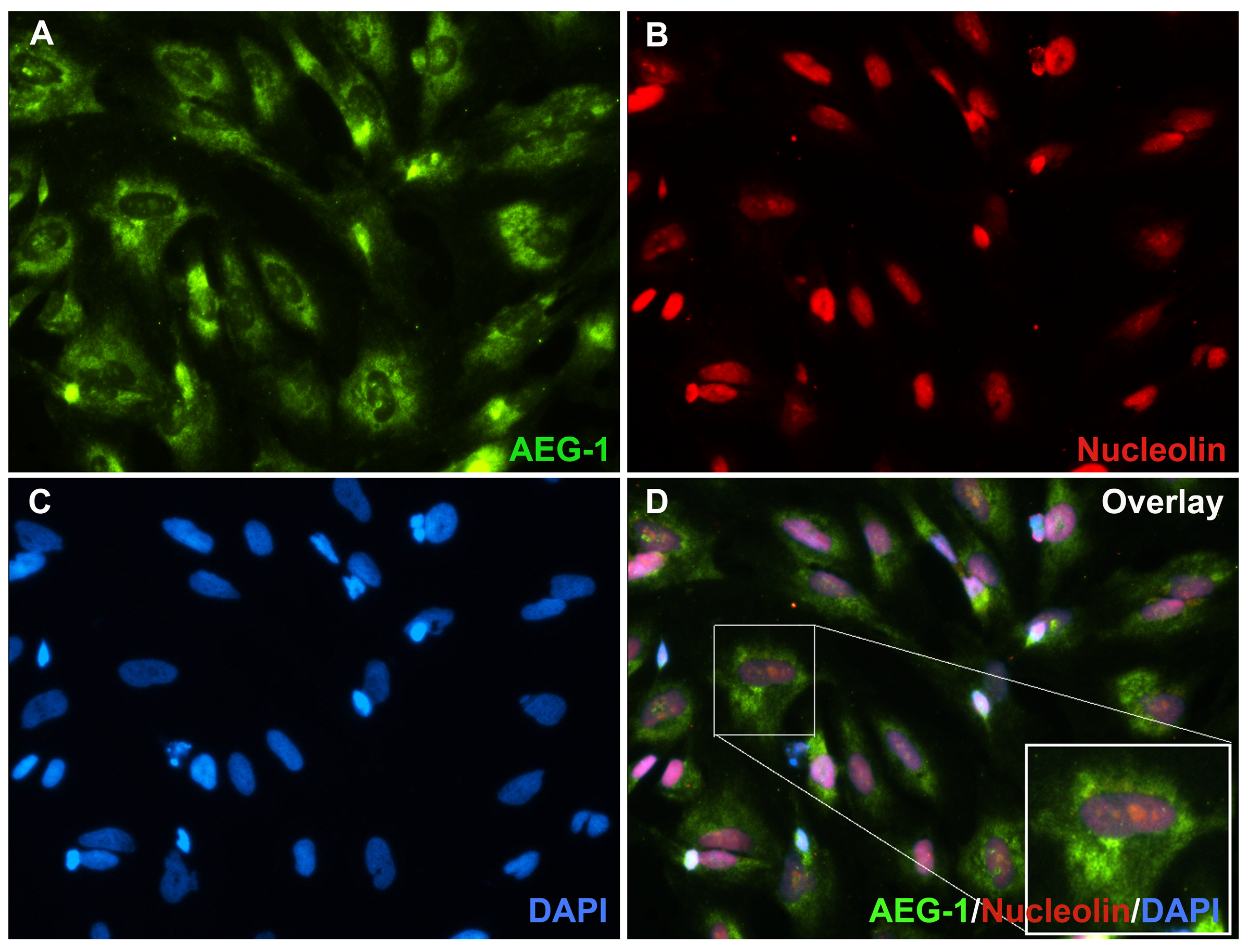

Supplement: Additional file 4 — Astrocyte elevated gene-1 (AEG-1) colocalizes with nucleolar protein nucleolin. At 48 h post injury astrocytes were immunostained for AEG-1 (green, (A)), nucleolin (red, nucleolar marker, 1:200, Abcam, (B)) and 4', 6-diamidino-2-phenylindole (DAPI) (blue, nuclear marker, (C)) and 20 × original magnification micrographs were overlaid (yellow, inset, (D)). Representative data from two individual donors assayed in triplicate. [file 1742-2094-9-195-S4.tiff]
